# Improving nelarabine efficacy in T cell acute lymphoblastic leukemia by targeting aberrant PI3K/AKT/mTOR signaling pathway

**DOI:** 10.1186/s13045-016-0344-4

**Published:** 2016-10-24

**Authors:** Annalisa Lonetti, Alessandra Cappellini, Alice Bertaina, Franco Locatelli, Andrea Pession, Francesca Buontempo, Camilla Evangelisti, Cecilia Evangelisti, Ester Orsini, Laura Zambonin, Luca Maria Neri, Alberto Maria Martelli, Francesca Chiarini

**Affiliations:** 1 Department of Biomedical and Neuromotor Sciences, University of Bologna, Bologna, Italy; 2 Department of Human Social and Health Sciences, University of Cassino, Cassino, Italy; 3 Department of Pediatric Hematology-Oncology, IRCCS, Bambino Gesù Children’s Hospital, Rome, Italy; 4 Department of Pediatrics, “Lalla Seràgnoli” Hematology-Oncology Unit, University of Bologna, Bologna, Italy; 5 Institute of Molecular Genetics, Rizzoli Orthopedic Institute, National Research Council, Bologna, Italy; 6 Department of Pharmacy and Biotechnology, University of Bologna, Bologna, Italy; 7 Department of Morphology, Surgery and Experimental Medicine, University of Ferrara, Ferrara, Italy

**Keywords:** PI3K signaling, Apoptosis, Drug resistance, Combination therapy

## Abstract

**Background:**

Although in recent years, the introduction of novel chemotherapy protocols has improved the outcome of T cell acute lymphoblastic leukemia (T-ALL) patients, refractory and/or relapsing disease remains a foremost concern. In this context, a major contribution was provided by the introduction of the nucleoside analog nelarabine, approved for salvage treatment of T-ALL patients with refractory/relapsed disease. However, nelarabine could induce a life-threatening, dose-dependent neurotoxicity. To improve nelarabine efficacy, we have analyzed its molecular targets, testing selective inhibitors of such targets in combination with nelarabine.

**Methods:**

The effectiveness of nelarabine as single agent or in combination with PI3K, Bcl2, and MEK inhibitors was evaluated on human T-ALL cell lines and primary T-ALL refractory/relapsed lymphoblasts. The efficacy of signal modulators in terms of cytotoxicity, induction of apoptosis, and changes in gene and protein expression was assessed by flow cytometry, western blotting, and quantitative real-time PCR in T-ALL settings.

**Results:**

Treatment with nelarabine as a single agent identified two groups of T-ALL cell lines, one sensitive and one resistant to the drug. Whereas sensitive T-ALL cells showed a significant increase of apoptosis and a strong down-modulation of PI3K signaling, resistant T-ALL cells showed a hyperactivation of AKT and MEK/ERK1/2 signaling pathways, not caused by differences in the expression of nelarabine transporters or metabolic activators. We then studied the combination of nelarabine with the PI3K inhibitors (both pan and dual γ/δ inhibitors), with the Bcl2 specific inhibitor ABT199, and with the MEK inhibitor trametinib on both T-ALL cell lines and patient samples at relapse, which displayed constitutive activation of PI3K signaling and resistance to nelarabine alone. The combination with the pan PI3K inhibitor ZSTK-474 was the most effective in inhibiting the growth of T-ALL cells and was synergistic in decreasing cell survival and inducing apoptosis in nelarabine-resistant T-ALL cells. The drug combination caused AKT dephosphorylation and a downregulation of Bcl2, while nelarabine alone induced an increase in p-AKT and Bcl2 signaling in the resistant T-ALL cells and relapsed patient samples.

**Conclusions:**

These findings indicate that nelarabine in combination with PI3K inhibitors may be a promising therapeutic strategy for the treatment of T-ALL relapsed patients.

**Electronic supplementary material:**

The online version of this article (doi:10.1186/s13045-016-0344-4) contains supplementary material, which is available to authorized users.

## Background

T cell acute lymphoblastic leukemia (T-ALL) is a hematologic malignancy resulting from the transformation of T cell progenitors that accounts for 15 % of pediatric and 25 % of adult ALL cases. Despite improvements in cure rates, the outcome of T-ALL patients with chemoresistant or relapsed leukemia is still poor [1]. T-ALL requires aggressive chemotherapy. To minimize and overcome the detrimental effects of therapeutic regimens, it is essential to identify novel molecular targets in T-ALL and test selective inhibitors of such targets [2]. Thus, major efforts are being made to develop targeted molecules against deregulated signaling pathways sustaining T-ALL cell growth/survival/drug resistance. Indeed, selective inhibitors of deregulated pathways could be used together with chemotherapy, allowing for a lower dosage of chemotherapeutic drugs which should minimize toxic side effects. The nucleoside analog nelarabine is a prodrug of the deoxyguanosine analog 9-β-d-arabinofuranosylguanine (Ara-G) [3, 4]. Nelarabine is rapidly metabolized in the plasma by an adenosine deaminase into the active metabolite Ara-G, the latter having a much longer plasma half-life and reaching higher plasma concentrations [5]. Ara-G is taken up by leukemia cells via the nitrobenzylthioinosine-sensitive nucleoside membrane transporter equilibrative nucleoside transporter 1 (ENT1) [6]. Ara-G is then phosphorylated to Ara-G monophosphate by the deoxycytidine and deoxyguanosine kinase (dCK and dGK, respectively). It is then phosphorylated to its triphosphate form Ara-GTP, which in turn competes with dGTP for DNA polymerase and is subsequently incorporated into DNA, resulting in termination of DNA synthesis [7]. Since 2005, nelarabine has been approved for the treatment of both pediatric and adult T-ALL patients who have refractory or progressive disease after previous chemotherapy regimens [5, 8]. Nelarabine is preferentially cytotoxic to T lymphoblasts through the accumulation of Ara-GTP, which occurs to a greater extent in T cells than in B cells, resulting in inhibition of ribonucleotide reductase and subsequent DNA synthesis [9, 10]. Nelarabine as monotherapy was effective in inducing complete/partial responses in both children and adults with refractory or relapsed T-ALL [3, 8, 11]. Given the impressive single-agent activity seen in refractory or relapsed T-ALL, substantial interest developed in evaluating nelarabine in the up-front treatment of T-ALL. Clinical trials have shown that nelarabine could be safely combined with intensive chemotherapy (BFM-86) in the frontline therapy of pediatric T-ALL, and in combination with hyper-CVAD in the frontline therapy for adults with T-ALL [12–14]. Of 40 patients with T-ALL or T lymphoblastic lymphoma (T-LL), the CR rate was 89 % in T-ALL and 94 % in T-LL. Overall survival at 3 years was 63 % [14]. However, in recent years, it is emerging that nelarabine could induce a significant neurotoxicity, particularly when given in conjunction with multi-agent chemotherapy regimens [15, 16]. Patients who suffered from nelarabine neurotoxicity displayed myelopathy and severe necrotic changes in the nervous system [17], as well as irreversible paresthesia or paraplegia [17, 18]. Importantly, nelarabine-induced neurotoxicity depends on the dosage [19]. Therefore, a combined therapy with signal transduction modulators could allow for the use of a lower dosage of nelarabine, resulting in a lower incidence and/or seriousness of neurotoxicity and resistance. However, there is no information regarding how aberrantly activated signaling pathways could influence T-ALL cell sensitivity to nelarabine. In contrast, in recent years, valuable information has been collected regarding aberrantly activated signaling pathways influencing leukemia cell sensitivity to fludarabine [20], a nucleoside analog which is employed in the treatment of B cell chronic lymphocytic leukemia (CLL), or to clofarabine, a nucleoside analog used for treating acute myeloid leukemia (AML) patients [21, 22]. The effects of phosphoinositide 3-kinase (PI3K) inhibition on fludarabine sensitivity could be related to changes in the expression of Bcl2 family proteins (Mcl-1 and Bim) [23, 24] which are also involved in clofarabine sensitivity in AML cells [22]. Importantly, PI3K/AKT/mammalian target of rapamycin (mTOR) signaling inhibition enhanced fludarabine-induced cell death in a T-ALL cell line (CEM-S) [25]. Moreover, a combination of clofarabine and temsirolimus, an mTOR inhibitor, displayed synergistic cytotoxic effects in AML cell lines and primary samples, and showed an encouraging clinical activity [21, 26]. A recent study underscored a potential role for Bcl2 family proteins in determining nelarabine resistance, in an Ara-G-resistant T-ALL cell line variant, established by serial incubation with Ara-G (the active metabolite of nelarabine) [27]. In this study, anti-apoptotic Bcl-xL was augmented and pro-apoptotic Bax and Bad were reduced in CEM/Ara-G cells, suggesting refractoriness to Ara-G-induced apoptosis [27].

Here, we demonstrate that in nelarabine-resistant T-ALL cell lines, there is a hyperactivation of PI3K/AKT/mTOR, ERK1/2 and Bcl2 signaling in response to nelarabine. Treatment with ZSTK-474 (a pan PI3K p110 inhibitor [28]), IPI-145 (Duvelisib^@^, a γ/δ PI3K p110 inhibitor [29]), GSK1120212 (Trametinib^@^, a MEK inhibitor [30]), or ABT199 (Venetoclax^@^, a Bcl2 inhibitor [31]) synergized with nelarabine in reducing cell survival in nelarabine-resistant T-ALL cells. ZSTK-474 was the most potent drug in inducing cell death in combination with nelarabine, allowing for a lower dosage of nelarabine even in samples from T-ALL relapsed patients. Moreover, nelarabine combined with ZSTK-474 induced a dephosphorylation of AKT and ERK1/2 and induced an increase in the expression of Bax and Bak pro-apoptotic members of Bcl2 family in T-ALL cells resistant to nelarabine. The combination of nelarabine with ZSTK-474 was able to induce a marked cell death in MOLT-4 cells co-cultured with HS-5 human HS-5 stromal cells, which mimic bone marrow (BM) microenvironment. These observations suggest the possibility to combine nelarabine together with selective inhibitors of the PI3K signaling pathway, to improve the efficacy of T-ALL treatment of relapsed/refractory patients.

## Results

### Treatment with nelarabine identifies two groups of T-ALL cell lines: one sensitive and one resistant

**Fig. 1:**
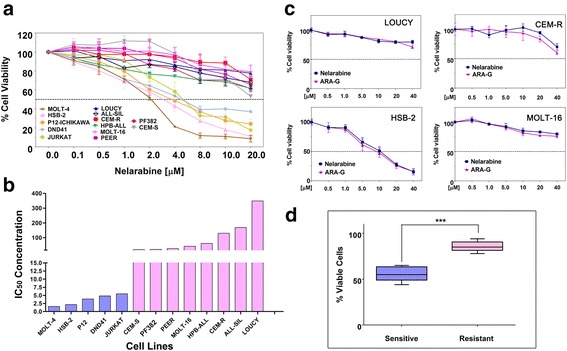
Nelarabine induces cytotoxic effects in T-ALL cell lines. **a** Cell viability was assessed by MTT analysis of T-ALL cell lines treated for 48 h with increasing concentrations of nelarabine. Results are the mean of at least three different experiments ± SD. **b** Identification of nelarabine sensitive and resistant cell lines based on IC_50_ values. **c** MTT analysis of T-ALL cell lines treated with nelarabine or Ara-G, at the same increasing concentrations for 48 h. Results are the mean of at least three different experiments ± SD. **d** Flow cytometric analysis of absolute cell count with eBeads123 and PI exclusion of T-ALL cells treated with nelarabine for 48 h. All 13 cell lines shown in Fig. 1a (five sensitive and eight resistant) were analyzed. Data were plotted and grouped. Results are the mean of three different experiments ± SD. *Asterisks* indicate statistically significant differences with respect to untreated cells (***P < 0.001)

The effects of nelarabine on a panel of T-ALL cell lines were tested by incubating the cells for 48 h with increasing concentrations of the drug and then analyzing the rate of cell viability, using MTT assays. Two significantly different groups of cell lines were outlined: a sensitive and a resistant group of cell lines. Cell viability decreased in a concentration-dependent fashion, and the IC_50_ values ranged between 2 and 5.5 μM for sensitive cell lines (Fig. 1a, b). However, it was observed that some cell lines were markedly resistant to nelarabine treatment. In particular, LOUCY, ALL-SIL, MOLT-16, and PEER cells did not reach an IC_50_ at the concentrations used, after 48 h of treatment (Fig. 1a, b). The most resistant cell line was LOUCY, with an IC_50_ of 300 μM, calculated with the CalcuSyn software. This cell line is representative of ETP-ALL, a T-ALL subtype with a very poor prognosis [29]. In vitro studies, using T-ALL cells, demonstrated that reduction of cell viability of induced by either nelarabine or Ara-G was comparable (Fig. 1c). Importantly, nelarabine induced a significant reduction in viable cell number, in sensitive versus resistant cell lines, as documented by absolute count (Fig. 1d).

### Nelarabine treatment promotes apoptosis in sensitive T-ALL cell lines

**Fig. 2:**
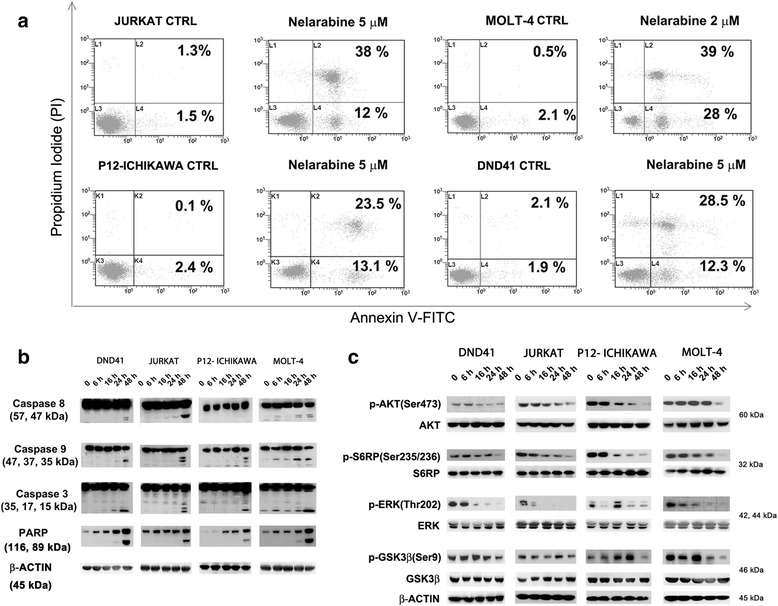
Nelarabine induces apoptosis in a group of sensitive T-ALL cell lines and modulates PI3K/AKT/mTOR and MEK signaling. **a** Flow cytometric analysis of Annexin V-FITC/PI stained T-ALL cells treated with nelarabine (5 μM for JURKAT, P12-ICHIKAWA, and DND-41 cells, 2 μM MOLT-4 cells) 48 h. The percentages of early apoptotic cells (Annexin-V FITC^+^/PI^−^; *bottom right quadrant*) and late apoptotic/necrotic cells (Annexin-V FITC^+^/PI^+^; *top right quadrant*) are plotted. The histograms are representative of three separate experiments. *CTRL* untreated cells. **b** Western blot analysis documenting cleavage of caspase-8, caspase-9, caspase-3, and PARP by nelarabine. Cells were treated with nelarabine (5 μM for JURKAT, P12-ICHIKAWA, and DND-41 cells, 2 μM MOLT-4 cells) for the indicated times, collected, and then lysed. Fifty micrograms of each lysate were electrophoresed on SDS-PAGE gels followed by transfer onto a nitrocellulose membrane. **c** Nelarabine induces a decrease in the phosphorylation status of critical components of the PI3K/AKT/mTOR signaling pathway, as well as p-ERK (Thr202) levels in T-ALL sensitive cell lines. Western blot analysis documenting the reduction of p-AKT (Ser473), p-S6RP, p-GSK3β (Ser9), and p-ERK (Thr202). Antibody to β-actin served as a loading control. Molecular weights are indicated on the right

To evaluate whether the effects of nelarabine on cell viability could be related to apoptosis, flow cytometric analysis was performed on the sensitive cell lines. In response to treatment with 5 μM nelarabine (2 μM for MOLT-4 cells), we detected a marked increase in the percentage of early apoptotic (positive for Annexin V) and/or late apoptotic (positive for both Annexin V and PI) cells after 48 h of treatment of T-ALL cell lines MOLT-4, JURKAT, P12-ICHIKAWA, and DND41 (Fig. 2a). Apoptosis was further investigated by western blotting, which documented a time-dependent cleavage of caspase 8, caspase 9, caspase 3, and poly(ADP-ribose) polymerase (PARP) in response to nelarabine treatment (Fig. 2b). All cell lines analyzed display phosphorylated AKT, which is indicative of constitutive activation of PI3K signaling pathway [32]. We examined the effects of nelarabine on the most important signaling downstream targets of PI3K/AKT/mTOR and MEK pathways. In all T-ALL sensitive cell lines, nelarabine treatment induced a marked decrease of phosphorylated AKT at Ser473, S6 ribosomal protein (S6RP) at Ser235/236, and GSK3β at Ser9 after 48 h of treatment, suggesting that PI3K/AKT/mTOR pathway is down-modulated by nelarabine (Fig. 2c). Moreover, in all sensitive cell lines analyzed, ERK phosphorylation was impaired by the treatment, indicating also a downregulation of the MEK pathway. In all cell lines evaluated, total protein levels were unaffected. It was demonstrated that DNA fragmentation is associated with nucleoside analog-induced apoptosis [27, 33]. To better understand the mechanism of nelarabine-induced apoptosis, we have analyzed the phosphorylation of H2AX. Indeed, the rapid phosphorylation of H2AX on Ser139 (γH2AX) is an early cellular response to double-strand breaks (DSBs). This phosphorylation event is one of the most well-established chromatin modifications linked to DNA damage. Moreover, the production of reactive oxygen species (ROS) coincides with the appearance of DNA damage and partially contributes to the DNA damage accumulated, followed by apoptosis at later time points [34]. MOLT-4 and Jurkat cell lines were treated with nelarabine for 7 and 24 h, and γH2AX was evaluated by flow cytometry in both control and treated cells. We observed a time-dependent increase of γH2AX in response to nelarabine in both cell lines compared to controls (untreated cells). In addition, we measured the intracellular ROS levels in response to nelarabine treatment, and we observed an increase of ROS production following 1 and 24 h of nelarabine treatment. The induction of DNA damage by nelarabine is consistent with a reduction in cell viability and an increase in caspases/PARP activity. These findings suggest that nelarabine-induced apoptosis could depend on these mechanisms (Additional file 1: Figure S1).

### Resistance to nelarabine is not dependent on differential expression of ENT1/2 transporters and is partly due to upregulation of PI3K/AKT/mTOR, MEK, and Bcl2 signaling

**Fig. 3:**
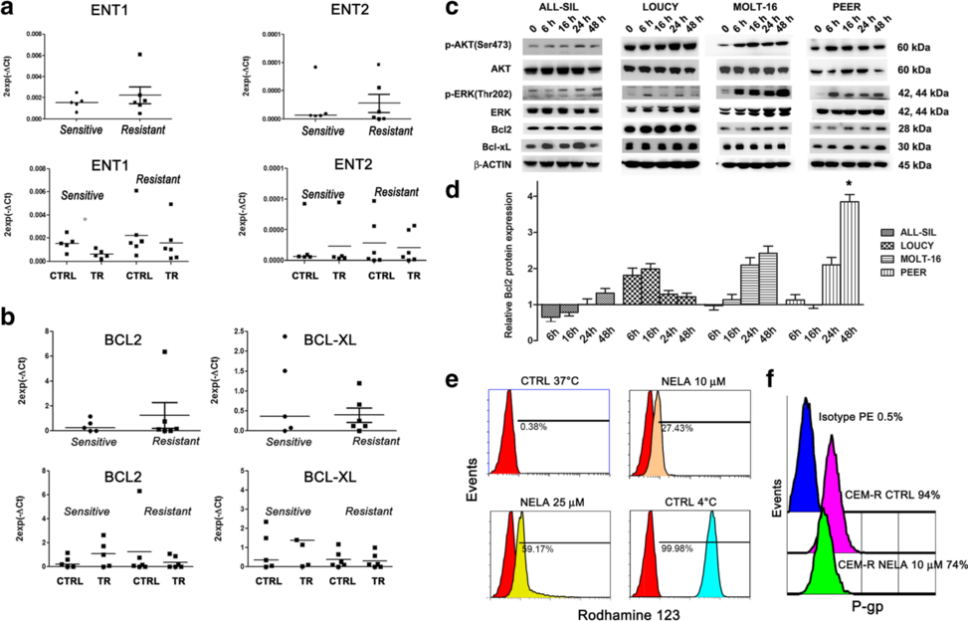
Nelarabine resistance does not depend on expression of ENT1/2 transporters and is partly due to upregulation of PI3K, MEK, and Bcl2 signaling. **a** Gene qRT-PCR analysis for ENT1 and ENT2 mRNA expression in T-ALL cell lines, untreated or treated with nelarabine for 48 h. Results are the mean from three different experiments ± SD. **b** qRT-PCR analysis for Bcl2 and Bcl-xL mRNA expression in T-ALL cell lines, untreated or treated with nelarabine for 48 h. Results are the mean from three different experiments ± SD. **c** Western blotting documenting an increase of p-AKT (Ser473), as well as p-ERK (Thr202), and Bcl2 in T-ALL resistant cell lines treated with nelarabine. Antibody to β-actin served as a loading control. **d** Densitometric analysis of western blotting shown in **c** was performed to quantify Bcl2 protein in resistant cell lines treated with nelarabine at different time points. The amount of protein was normalized to β-actin density and expressed as fold change compared to control (ratio = Bcl2 treated/Bcl2 control). Densitometry scanning of the bands was performed using a Chemidoc 810 Imager with the appropriate software (UVP, Upland, CA, USA). Statistical analyses were performed with the Dunnett’s multiple comparison test. Results showed a significant increase in the Bcl2 protein expression only in PEER cell line, at 48-h treatment, *P* < 0.05. **e** Flow cytometric functional efflux activity assay for 170 kDa P-glycoprotein (P-gp) in CEM-R (overexpressing P-gp) cells. The assay is based on extrusion of the fluorescent P-gp substrate, Rhodamine 123. The efflux activity of P-gp is highly temperature sensitive; P-gp functions optimally at 37 °C, but is inactive at 4 °C. When preloaded with Rhodamine 123 and incubated at 4 °C (CTRL), CEM-R cells retained the dye and consequently exhibited high fluorescence. Conversely, cells incubated at 37 °C (CTRL) effluxed the dye. CEM-R cells treated with nelarabine at 37 °C displayed a marked decrease in the pump activity. **f** Flow cytometric analysis of surface P-gp expression of CEM-R cells treated with nelarabine for 48 h (10 μM). The histograms are representative of two separate experiments

To find potential explanations for differences in nelarabine sensitivity displayed by T-ALL cell lines, we determined mRNA expression levels of ENT1 and ENT2 nelarabine transporters, which could have a role in nelarabine cellular uptake [35]. Both ENT1 and ENT2 were expressed in all T-ALL cell lines, but there were no differences between the sensitive versus resistant group in the levels of expression of these transporters (Fig. 3a). Moreover, nelarabine treatment did not affect ENT1/2 mRNA levels in T-ALL sensitive or resistant groups (Fig. 3a). By western blotting, we have also evaluated the expression of the two enzymes, dCK and dGK, involved in the purine metabolism. However, no significant differences in the expression of these enzymes in sensitive versus resistant group were detected (Additional file 2: Figure S2).

A recent study underscored a potential role for Bcl2 family members in determining nelarabine resistance, as a nelarabine-resistant T-ALL cell line displayed increased anti-apoptotic Bcl-xL levels, whereas pro-apoptotic Bax and Bad levels were decreased [27]. To better understand the role of Bcl2 family, it was first investigated the gene expression of Bcl2 and Bcl-xL genes in sensitive and resistant to nelarabine cell lines. Analyses of gene expression demonstrated no significant differences in the levels of mRNA, between sensitive and resistant to nelarabine T-ALL cell lines under basal conditions (Fig. 3b, upper panel). Moreover, treatment with nelarabine did not induce significant differences in the gene expression of resistant or sensitive cell lines (Fig. 3b, lower panel). By western blotting, we analyzed the effects of nelarabine on the expression of anti-apoptotic Bcl2 and Bcl-xL proteins in resistant cell lines. Among T-ALL cell lines, LOUCY cell line showed the highest level of expression of Bcl2 under basal conditions (Additional file 3: Figure S3). Nelarabine treatment affected the expression of Bcl2 in all resistant cell lines analyzed. However, a significant increase of Bcl2 protein expression was observed only in PEER cells, after 48-h treatment (Fig. 3c, d).

Aberrant activation of several signal transduction pathways enhances survival, proliferation, and drug resistance of leukemic cells. PI3K/AKT/mTOR pathway is frequently overactive in T-ALL cells, and is very important for drug resistance. Therefore, it was investigated the status of this pathway in nelarabine resistance. T-ALL cell lines showing high IC_50_ to nelarabine treatment were treated for 6, 16, 24, 48 h with the drug (10 μM) and then analyzed by western blotting for the activation of PI3K/AKT/mTOR or MEK/ERK pathways. In ALL-SIL, LOUCY, MOLT-16, and PEER cell lines, treatment with nelarabine induced a time-dependent increase in p-AKT (Ser473), indicating an overactivation of PI3K/AKT signaling (Fig. 3c). It was also investigated the status of the MEK/ERK1/2 pathway in these resistant cell lines. This signaling cascade is also involved in leukemic cell survival and drug resistance [36]. Western blotting analysis documented that nelarabine dramatically increased p-ERK (Thr202) levels in all resistant cell lines analyzed, especially in MOLT-16 and PEER cells (Fig. 3b). The results presented above indicated these signaling cascades as attractive targets for the development of new therapeutic strategies for T-ALL cells resistant to nelarabine treatment.

### Effects of nelarabine on 170-kDa P-glycoprotein

No information is at present available regarding the effects of 170-kDa P-glycoprotein (P-gp), one of the major determinants of multidrug resistance in acute leukemias [37], on nelarabine sensitivity.

In contrast, it is known that pharmacological inhibition of P-gp activity increased fludarabine sensitivity in CLL cells [38]. Furthermore, P-gp expression is known to be under the control of PI3K/AKT/mTOR signaling in acute leukemia cells [39, 40]. We noticed that CEM-R cells, which overexpress P-gp [41], were less sensitive to nelarabine than parental CEM-S cells. Therefore, we set out to investigate the relevance of P-gp in nelarabine sensitivity. A functional assay for P-gp activity, based on Rhodamine 123 extrusion, demonstrated that nelarabine treatment (48 h) was able to decrease P-gp activity in CEM-R cells (Fig. 3d). Moreover, flow cytometric analysis documented that nelarabine negatively affected P-gp surface expression in these cells (Fig. 3e). Therefore, our findings indicated that nelarabine actually decreased P-gp activity and expression in CEM-R cells. Thus, it is unlikely that the different sensitivity to nelarabine displayed by CEM-S and CEM-R cells could be dependent on P-gp expression.

### Nelarabine combined with ZSTK-474 affects PI3K/AKT signaling and modulates p-ERK and Bcl2 family members in T-ALL cells resistant to nelarabine

**Fig. 4:**
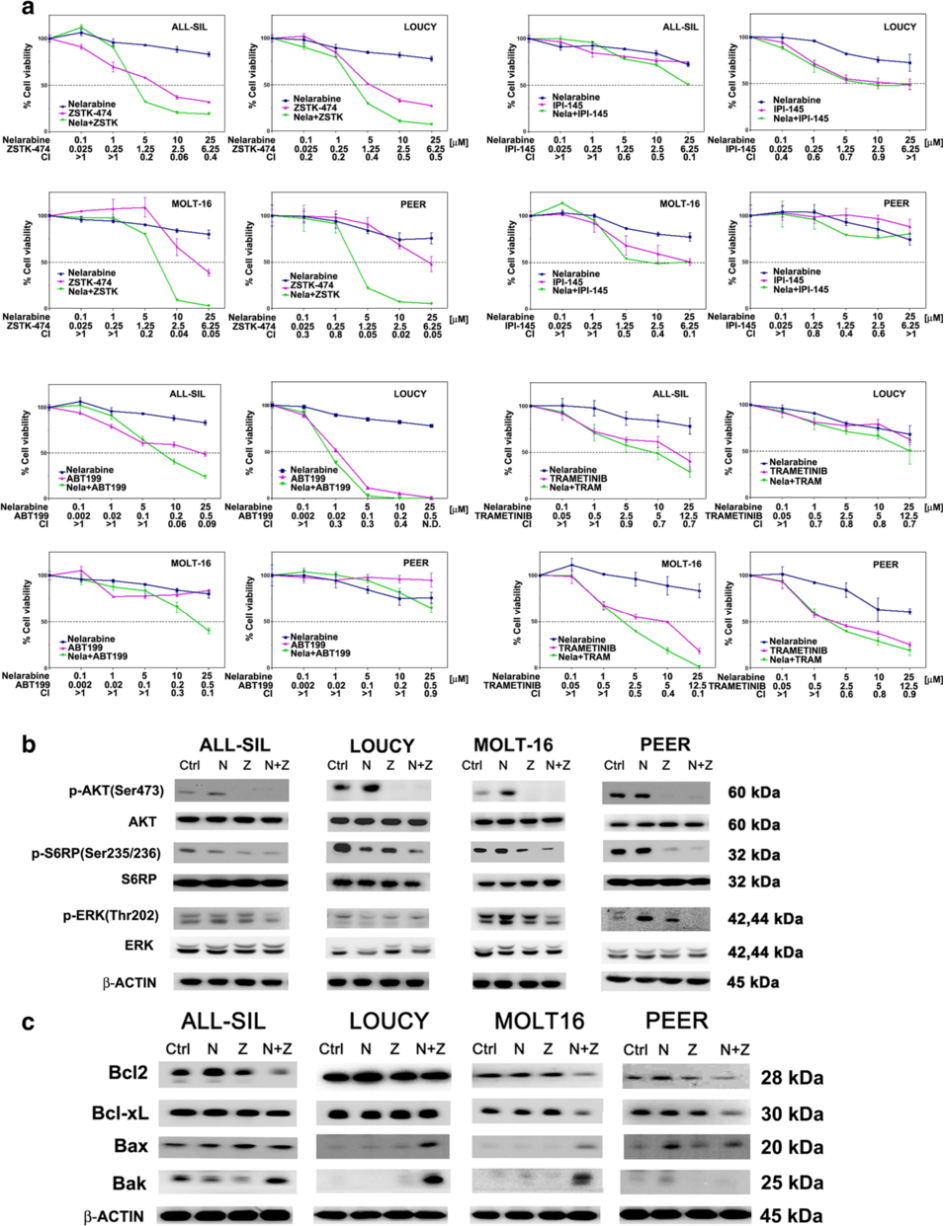
Nelarabine combined with the PI3K inhibitor ZSTK-474 induces cytotoxicity and causes a down-modulation of PI3K, MEK, and Bcl2 signaling. **a** Cell viability assays of T-ALL cell lines treated for 48 h with increasing concentrations of nelarabine combined with the pan PI3K p110 inhibitor ZSTK-474, the γ/δ PI3K p110 inhibitor IPI-145, the MEK inhibitor trametinib, or the Bcl2 inhibitor ABT199 at fixed ratios. Results are the mean of three different experiments ± SD. **b** Western blotting analyses documenting a marked decrease in the phosphorylation of AKT, S6RP, and ERK in T-ALL cell lines treated with the combination of nelarabine (10 μM) and ZSTK-474 (2.5 μM). Molecular weights are indicated on the *right*. One representative of three different experiments is shown. **c** Western blotting analyses of T-ALL cell lines treated with the combination of nelarabine (10 μM) and ZSTK-474 (2.5 μM) for 48 h. The combined treatment decreased the levels of expression of Bcl2 and Bcl-xL and increased the expression of the pro-apoptotic Bax and Bak proteins. Antibody to β-actin served as a loading control. Molecular weights are indicated on the *right*. One representative of three different experiments is shown

To better understand the role of PI3K, MEK/ERK1/2, and Bcl2 signaling, it was investigated the effect of the combination of nelarabine with the pan PI3K inhibitor ZSTK-474, the γ/δ PI3K p110 inhibitor IPI-145, the MEK inhibitor trametinib, or the Bcl2 inhibitor ABT199. The effects of the different drug combinations on nelarabine-resistant T-ALL cell lines were analyzed by treating the cells with increasing concentrations of the drugs and then measuring the rates of survival by MTT assays at 48 h. Cell lines (LOUCY, MOLT-16, PEER, and ALL-SIL) were cultured in the presence of nelarabine or the abovementioned drugs, either alone or in combination at a fixed ratio (Fig. 4a). The combined treatment with ZSTK-474 was highly effective in inducing cytotoxicity in all the resistant cell lines analyzed, indicating that PI3K is at least in part involved in nelarabine resistance. ZSTK-474 was synergistic with nelarabine also in P-gp overexpressing CEM-R cells (Additional file 4: Figure S4).

A prominent role for PI3K p110γ and δ isoforms has been proposed in T-ALL [42]. Therefore, the effects of the p110 γ/δ dual inhibitor IPI-145 alone or in combination with nelarabine were also assessed. However, the pan PI3K inhibitor ZSTK-474 was more efficacious than IPI-145 although IPI-145 combined with nelarabine was also synergistic. The combination index (CI) values, calculated with CalcuSyn software for dose-effect analysis, indicated the existence of a strong synergism for both drugs (CI <0.5) (Fig. 4a).

Treatment with the MEK inhibitor trametinib was also synergistic, but less potent in inhibiting cell viability in all cell lines studied. Interestingly, MOLT-16 and PEER cells, which displayed the most marked activation of MEK/ERK1/2 signaling after nelarabine treatment, were the most sensitive to trametinib. The Bcl2 inhibitor, ABT199, was very efficacious in inducing cell growth impairment, especially in ALL-SIL and LOUCY cell lines (Fig. 4a). LOUCY cells are representative of ETP-ALL and displayed the highest Bcl2 expression and sensitiveness to Bcl2 inhibition by ABT199 [43].

To better assess the role of PI3K signaling, western blot analyses with an antibody to p-AKT (Ser473) were carried out, demonstrating a marked decrease in response to the combination of nelarabine with ZSTK-474 treatment after 48 h, in all cell lines analyzed (Fig. 4b). Also, one of the downstream substrates of mTORC1 (S6RP) was efficiently dephosphorylated by the drug combination in all cell lines. Furthermore, the combination of nelarabine with ZSTK-474 was able to induce a decrease in the Bcl2 and Bcl-xL protein expression in all cell lines resistant to nelarabine alone, except for LOUCY cells, which were not affected by the combined treatment (Fig. 4c). The combination was also capable of inducing an increase in the expression of pro-apoptotic Bax and Bak proteins after 48 h of treatment (Fig. 4c).

To assess whether nelarabine directly affected PI3K/AKT and MEK/ERK1/2 signaling pathways, we have treated T-ALL resistant to nelarabine cells with selective inhibitors of these two axes. In particular, we employed LY294002 (PI3K inhibitor), CCI-779 (mTOR allosteric inhibitor), and trametinib (MEK1/2 inhibitor). As shown in Additional file 5: Figure S5, we confirmed that treatment with nelarabine as a single agent upregulated p-AKT (Ser473) and p-ERK (Thr202), whereas LY294002, CCI-779 and trametinib as single agents did not. LY294002 was not able to dephosphorylate AKT and to synergize with nelarabine, in contrast to the PI3K inhibitor ZSTK-474. This result could be dependent on the LY294002 concentration we used, 10 μM to avoid aspecific effects on signaling pathways, whereas published data employed LY294002 at higher concentrations [41]. Combining nelarabine with CCI-779 inhibitor resulted in upregulated PI3K and MEK signaling as demonstrated by an increase in p-AKT (Ser473) and p-ERK (Thr202), compared to untreated or CCI-779 treated cells, and indicating a major role of nelarabine in hyperactivating these axes. To ascertain CCI-779 effects, we evaluated one of its downstream key targets S6RP, which was strongly dephosphorylated by the treatment. Trametinib was able to completely dephosphorylate ERK both alone and in combination with nelarabine, but it did not perturbate significantly AKT phosphorylation. However, also in this case, the combination of trametinib with nelarabine increased p-AKT (Ser473) compared to T-ALL cells untreated or treated with nelarabine/trametinib as single agents. These results suggest that the upregulation of PI3K/AKT and MEK/ERK1/2 signaling pathways are directly affected by nelarabine treatment. Moreover, ZSTK-474 strongly counteracted nelarabine effects on both PI3K and MEK/ERK1/2 signaling pathways compared to the other specific inhibitors employed.

### The combination of nelarabine with ZSTK-474 restored sensitiveness in MOLT-4 and P12-ICHIKAWA cells co-cultured with of HS-5 stromal cells and down-modulated PI3K and Bcl2 signaling in a system which mimics BM microenvironment

**Fig. 5:**
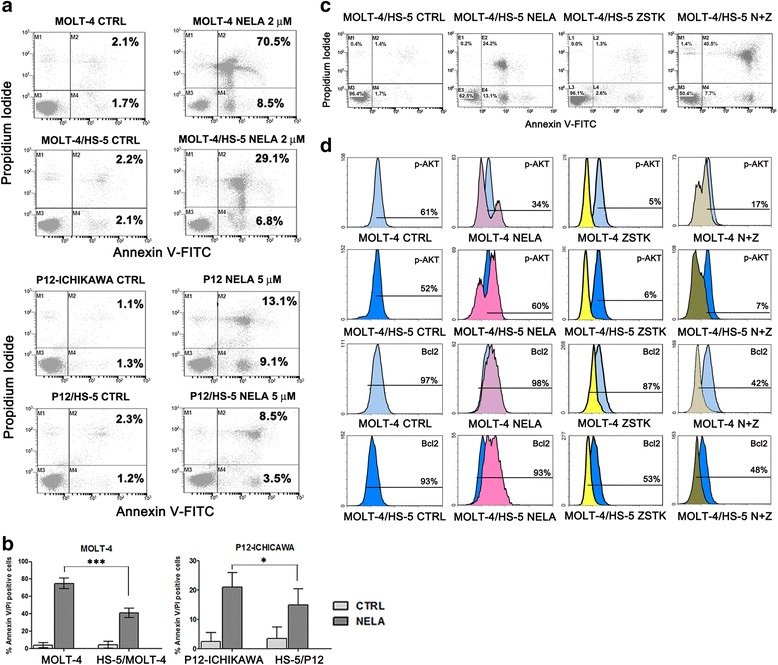
ZSTK-474 restored nelarabine sensitivity in MOLT-4 and P12-ICHIKAWA cells co-cultured with of HS-5 stromal cells and down-modulated PI3K and Bcl2 signaling. **a** Flow cytometric analysis of Annexin V-FITC/PI staining in T-ALL cells growing alone or in co-culture system with HS-5 cells and treated with nelarabine (5 μM for P12-ICHIKAWA cells, 2 μM for MOLT-4 cells) in a Transwell^@^ system. Three hundred seventy-five thousand T-ALL cells were seeded in the upper chamber of the Transwell^@^ system. The histograms are representative of three separate experiments. *CTRL* untreated cells. **b** *Graph bars* indicating apoptotic cells in response to combined treatment in single culture versus co-culture with HS-5 cells. *Asterisks* indicate statistically significant differences with respect to untreated cells (***P < 0.001, *P < 0.05). **c** Flow cytometric analysis of Annexin V-FITC/PI of MOLT-4 cells growing in co-culture with HS-5 stromal cells and treated with nelarabine 2 μM for 48 h in a Transwell^@^ system. The histograms are representative of three separate experiments. **d** Flow cytometric analysis of p-AKT (Ser473) and Bcl2 in MOLT-4 cells growing alone or in co-culture with HS-5 stromal cells. The histograms are representative of three separate experiments

We employed the human stromal cell line HS-5, known to provide long-term support for hematopoietic progenitors [44]. It was investigated whether cell death induced by nelarabine treatment in sensitive cell lines could be impaired by co-culturing T-ALL cells with HS-5, which mimic the BM microenvironment. MOLT-4 and P12-ICHIKAWA cells displayed a significant protection from apoptosis, induced by the co-culture with HS-5 stromal cells at 48 h of treatment, as demonstrated by staining with Annexin V/PI and flow cytometric analysis of apoptosis (Fig. 5a, b). The combination of nelarabine and ZSTK-474 was efficient in decreasing resistance to nelarabine, induced in these two cell lines by the co-culture with HS-5 stromal cells. Moreover, to better assess the effectiveness of a combined treatment, we examined MOLT-4 cells for the levels of p-AKT (Ser473) and Bcl2, with or without the support of HS-5 stromal cells, using flow cytometry. Flow cytometric analysis documented a decrease in the levels of p-AKT (Ser473) in nelarabine-treated samples without the support of stromal cells (Fig. 5c). Interestingly, nelarabine induced a hyperphosphorylation of AKT in MOLT-4 cells supported by the co-culture with HS-5 cells, while the treatment with ZSTK-474 was able to induce a marked reduction of p-AKT and Bcl2 expression. Overall, these findings demonstrated that the combination of nelarabine with ZSTK-474 has a potent cytotoxic activity also in co-culture conditions which mimic the BM microenvironment.

### HS-5 stromal cells protect MOLT-4 cells from nelarabine cytotoxicity at least in part through CXCL12/CXCR4 interactions

It is well established that interactions between the chemokine CXCL12, secreted by BM stromal cells, and its receptor CXCR4, expressed on the surface of leukemic cells, are very important for protecting leukemia cells from chemotherapeutic drugs [37, 45]. HS-5 cells secrete CXCL12 [46], while MOLT-4 cells express high levels of CXCR4 [47]. Therefore, we wanted to establish whether CXCL12/CXCR interactions were involved in the protective effects exerted by HS-5 cells on nelarabine-treated MOLT-4 cells. As shown in Additional file 6: Figure S6A, AMD3100 (plerixafor), which selectively blocks the interactions between CXCL12 and CXCR4 [47], significantly increased the cytotoxicity of nelarabine in MOLT-4 cells co-cultured with HS-5 cells in a Transwell^@^ system.

To document that the CXCR4/CXCL12 axis was indeed functional in our system, we performed western blot analysis. The presence of HS-5 cells increased p-ERK and p-CXCR4 levels in MOLT-4 cells, as expected [48]. It is worth noting that Ser339 is an amino acidic residue previously identified as a site for ligand-induced phosphorylation of CXCR4 [49] and is involved in ERK1/2 activation [50, 51]. The presence of nelarabine did not further increase the phosphorylation or either ERK or CXCR4. However, AMD3100 dampened the phosphorylation levels of both ERK and CXCR4 (Additional file 6: Figure S6B).

### T-ALL blasts are sensitive to the combination of nelarabine with ZSTK-474

**Fig. 6:**
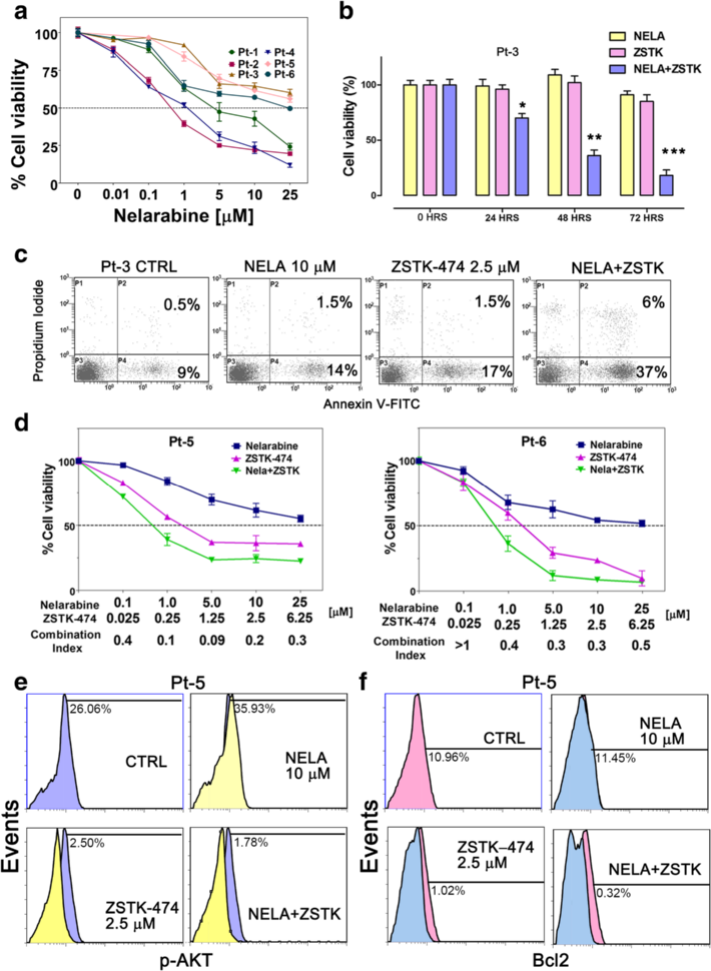
Nelarabine combined with ZSTK-474 is able to induce a marked cytotoxic effect in relapsed T-ALL patients with upregulated PI3K signaling. **a** MTT assays of lymphoblasts from relapsed T-ALL patients (Pts) with activated PI3K signaling treated with nelarabine for 72 h. Data shown are the mean of at least two different experiments ± SD. **b** Flow cytometric cell count with eBeads123/PI staining of T-ALL blasts treated with nelarabine alone or combined with ZSTK-474 for 24, 48,72 h. **c** Flow cytometric analysis of Annexin V-FITC/PI of T-ALL blasts treated with nelarabine alone or combined with ZSTK-474. The percentages of early apoptotic cells (Annexin-V FITC^+^/PI^−^; *bottom right quadrant*) and late apoptotic/necrotic cells (Annexin-V FITC^+^/PI^+^; *top right quadrant*) are plotted. The histograms are representative of two separate experiments. **d** Cell viability assays of lymphoblasts from relapsed T-ALL patients treated for 72 h with increasing concentrations of nelarabine alone or combined with the pan PI3K inhibitor ZSTK-474 at fixed ratios. Results are the mean of two different experiments ± SD. **e**, **f** Flow cytometric analysis documenting a decrease of p-AKT (Ser473) (**e**) and Bcl2 (**f**) levels, respectively, in response to the combination of nelarabine and ZSTK-474 at the indicated concentrations for 48 h. The histograms are representative of two separate experiments

To better assess the effectiveness of the combination of nelarabine with ZSTK-474 as a potential therapeutic strategy in T-ALL, we examined relapsed pediatric T-ALL patient samples isolated from the bone marrow or peripheral blood, for the sensitiveness to nelarabine alone, in vitro. These patients were previously analyzed for the activation of PI3K signaling, showing high levels of p-AKT (Ser 473). The effects of nelarabine on patient lymphoblasts were tested by incubating the cells for 72 h with increasing concentrations of the drug and then analyzing the rate of cell viability using MTT assays. Some patient samples showed high IC_50_ (>10 μM), while others were more sensitive to nelarabine alone (IC_50_ <5 μM) (Fig. 6a). The resistant samples were treated with nelarabine in combination with ZSTK-474, and absolute cell count/PI staining was assessed by flow cytometry for 24, 48, and 72 h for Pt-3, showing the significant effects of the combination (Fig. 6b). The combination of nelarabine with ZSTK-474 was highly effective in inducing apoptosis in the resistant patient samples analyzed, showing a marked increase in Annexin V/PI stained (Fig. 6c, Pt-3). Pt-5 and Pt-6 were also evaluated by MTT assays with nelarabine in combination with ZSTK-474 at fixed ratios (nelarabine/ZSTK-474, 4:1). The synergistic effect was assessed and the combination indexes (CIs) were calculated with the CalcuSyn software (Fig. 6d).

Finally, we evaluated the effects of single treatment with nelarabine or combination with ZSTK-474 on T-ALL patient samples on the expression levels of p-AKT (Ser473) and Bcl2. Flow cytometric analysis documented an increase in the levels of p-AKT in nelarabine-treated samples, while the treatment with the combination of nelarabine and ZSTK-474 was able to induce a marked reduction of p-AKT (Ser473) and Bcl2 expression (Fig. 6e, f). Overall, these findings demonstrated that the combination of nelarabine with ZSTK-474 has a potent cytotoxic activity also in primary cells from relapsed T-ALL patients with upregulated PI3K/AKT signaling and resistant to nelarabine in vitro.

## Discussion

Despite the continuous efforts in understanding the molecular complexity of T-ALL, treatments are still based on chemotherapeutic regimens, and prognosis of this disease remains poor, especially in the adult and in chemoresistant/relapsed patients. Nelarabine is a nucleoside analog successfully used for the treatment of refractory/relapsed T-malignancies, including T-ALL and T-LL [52–54]. Nelarabine as monotherapy is effective in inducing complete/partial responses in T-ALL patients [5]. However, it could induce a severe dose-dependent neurotoxicity. Very little is known of proteins/genes that could influence nelarabine sensitivity in T-ALL [35, 55]. Here, we evaluated the in vitro efficacy of the administration of nelarabine as a single agent in cell lines and primary blast cells from relapsed T-ALL, to explore some druggable signaling pathways that influence sensitivity to nelarabine. Indeed, a combined therapy with signal transduction modulators could allow for the use of a lower dosage of nelarabine, resulting in a lower incidence and/or seriousness of neurotoxicity and major effects of the therapy. Importantly, our in vitro studies, using T-ALL cell lines, demonstrated that IC_50_ values of nelarabine and Ara-G are comparable. The concentrations utilized in these experiments are lower than plasma levels achievable in Ara-G-treated patients [56]. We have shown that nelarabine reduced cell viability in a concentration-dependent manner in a group of T-ALL cell lines and primary patient samples, with an IC_50_ lower than 5 μM, while other cell lines and relapsed patients showed resistance to in vitro nelarabine treatment, with IC_50_ much higher than 10 μM. The nelarabine cytotoxic effects in sensitive cell lines correlated with a significant induction of apoptosis and cleavage of caspases 8, 9, and 3, and PARP, suggesting the activation of the extrinsic and intrinsic apoptotic pathway. Moreover, PI3K signaling and MEK/ERK1/2 pathway resulted down-modulated in all the sensitive cell lines treated with nelarabine alone. It has been reported that the levels of expression of ENT1/2 nucleoside transporters were related to in vitro nelarabine sensitivity of T-ALL cell lines and primary samples [35]. These transporters have been shown to be important for the Ara-C import, and elevated ENT1 levels have been reported to explain the high Ara-C sensitivity of infant ALL [57]. However, a previous work on the T-ALL cell line CEM-S demonstrated that, while the cellular transport of forodesine was dependent on ENT1/2, forodesine toxicity was not [58]. Recent studies also showed that ENT1/2 expression levels were not related to forodesine toxicity levels in T-ALL cells [35]. We have analyzed the expression of ENT1/2 transporters in the sensitive and resistant to nelarabine groups. In all the T-ALL cells analyzed, we did not find significant differences between resistant and sensitive cells as far as the expression of ENT1/2 transporters was concerned. Furthermore, the levels of expression of ENT1/2 genes were not modulated by the treatment with nelarabine in a significant manner. In addition, Ara-G must be phosphorylated by dCK and dGK to become an intracellular metabolite and to be incorporated into DNA [7]. The protein levels of dCK and dGK were not significantly different in resistant or sensitive groups, indicating that the resistance to nelarabine in T-ALL cells could be due to other mechanisms. Accumulating evidence indicates that the PI3K signaling is linked to resistance to therapy in several disorders [59, 60]. In hematological malignancies, it has been shown to support tumor proliferation, viability, and drug resistance. In a recent paper by Silveira et al. [61], it was shown that PI3K pathway activity was higher in T-ALL patients who underwent relapse [61]. Owing to the fundamental role of PI3K pathway in tumors and in particular in T-ALL, we investigated the phosphorylation status of AKT, a direct downstream target of PI3K, in T-ALL cells. We have shown that in resistant T-ALL cell lines and primary relapsed T-ALL blasts, nelarabine alone caused an increase of AKT phosphorylation at Ser473. Accordingly, PI3K inhibition with ZSTK-474 deeply sensitized T-ALL cells to nelarabine, while the combination of nelarabine with either MEK or Bcl2 inhibitors was less synergistic in inducing cell death in T-ALL cell lines. Because at present, it is impossible to predict if patient could benefit from nelarabine treatment, this study for the first time uncovered mechanisms responsible for resistance to nelarabine in T-ALL cells, paving the way to novel combination strategies, able to overcome resistance and facilitate the bridge to hematopoietic cell transplantation. Indeed, these findings strongly suggest that combining nelarabine with inhibitors of PI3K/AKT/mTOR signaling may represent a possible strategy for the treatment of relapsed or refractory patients with T-ALL. In this sense, it should be emphasized that a recent phase I/II clinical trial has demonstrated that the mTOR inhibitor everolimus was moderately effective in combination with hyper-CVAD chemotherapy especially in relapsed/refractory T-ALL patients [45].

## Conclusions

Therefore, data obtained from this work could improve the therapy of refractory/relapsed T-ALL through the design of new regimens that could ameliorate clinical care of patients and their outcome, providing more effective interventions for patients that are resistant to the current therapies.

## Methods

### Materials

Nelarabine, ZSTK-474, IPI-145, trametinib, CCI-779, and ABT199 were kindly provided by Selleckchem (Houston, TX, USA). Ara-G, insulin, transferrin, sodium selenite (ITS), propidium iodide (PI), AMD3100, and LY294002 were purchased from Sigma-Aldrich (St. Louis, MO, USA). For western blotting analysis, all the primary antibodies were purchased from Cell Signaling Technology (Danvers, MA, USA) except for the antibodies to CXCR4 and p-CXCR4 (Ser339) which were from Abcam (Cambridge, UK) and antibodies to dGK and dCK which were from Thermo Scientific (Thermo Scientific, Waltham, MA, USA). AlexaFluor^@^-conjugated antibodies were from Cell Signaling Technology or BD Biosciences (Franklin Lakes, NJ, USA).

### Cell cultures and patient samples

A panel of human T-ALL cell lines (HPB-ALL, DND41, RPMI-8402, JURKAT, MOLT-4, MOLT-16, CEM-S, CEM-R drug resistant, ALL-SIL, LOUCY, HSB-2, PEER, KOPTK1, PF382, P12-ICHIKAWA) was employed. HS-5 human stromal cells were also employed to mimic bone marrow microenvironment. All cell lines, except HS-5, were from Deutsche Sammlung von Mikroorganismen und Zellkulturen GmbH (DSMZ, Braunschweig, Germany). HS-5 cells were from ATCC (Manassas, VA, USA). Primary blast cells from relapsed T-ALL were obtained, upon written informed consent in accordance with the Declaration of Helsinki and the study has been approved by the ethics committee (Independent Ethics Committee of the University Hospital of Bologna “S. Orsola-Malpighi”). Blasts from the bone marrow and peripheral blood samples were obtained by density gradient centrifugation over Lymphoprep (Nycomed UK, Birmingham, UK). Cell cultures and primary T-ALL refractory/relapsed lymphoblasts were grown in RPMI 1640 + ITS, supplemented with 10 or 20 % fetal bovine serum (FBS), l-glutamine, and penicillin–streptomycin. HS-5 human stromal cells were grown in MEM Alpha Modification (Sigma-Aldrich) medium supplemented with 10 % FBS, l-glutamine, and penicillin–streptomycin.

### Cell viability analysis

MTT (3-[4,5-dimethylthythiazol-2-yl]-2,5-diphenyltetrazolium bromide) assays were performed to assess the sensitivity of cells to drugs, as previously reported [62].

### Annexin V-fluorescein isothiocyanate (FITC)/PI staining

To assess the extent of apoptosis induction, a flow cytometric analysis of Annexin V-FITC/PI-stained samples was performed. Analyses were performed on a FC500 flow cytometer (Beckman, Miami, FL, USA), with the appropriate software (CXP, Beckman). At least 15,000 events per sample were acquired.

### Quantification of p-H2AX (Ser139) (γH2AX) by flow cytometry

Cells were treated with nelarabine (2 μM MOLT-4; 5 μM Jurkat) and fixed in 70 % ethanol over night at −20 °C. Afterwards, cells were rehydrated for 10 min at 4 °C in PBS containing 4 % FBS and 0.1 % Triton X-100. Permeabilized cells were incubated with an Alexa Fluor 647 Mouse anti p-H2AX (Ser139) (BD Bioscience) at room temperature. Fluorescence was measured by flow cytometer on a FC500 flow cytometer (Beckman, Miami, FL, USA), with the appropriate software (CXP, Beckman). At least 10,000 events per sample were acquired.

### Measurement of intracellular reactive oxygen species (ROS) levels

ROS intracellular level was evaluated by using the fluorescent probe 2′,7′-dichlorodihydrofluorescein diacetate (H_2_DCFDA). Cells (0.5 × 10^6^/mL) were incubated with nelarabine for 1 and 24 h; then, cells were washed twice in HBSS and incubated with 5 μM H_2_DCFD for 20 min at 37 °C. H_2_DCFDA is a small non-polar, non-fluorescent molecule that diffuses into the cells, where it is enzymatically deacetylated by intracellular esterases to a polar non-fluorescent compound, which is oxidized to the highly green fluorescent 2′,7′-dichlorofluorescein (DCF). The fluorescence of oxidized probe was measured using a multiwell plate reader (Wallac Victor^2^, PerkinElmer). Excitation wavelength was 485 nm, and emission wavelength was 535 nm. Fluorescence values were reported as the percentage of intracellular ROS in respect to controls.

### Western blot analysis

This was performed by standard methods, as previously reported [62].

### Bcl2, Bcl-xL, and ENT1/2 expression analyses by qRT-PCR

Total RNA was extracted using the RNeasy Mini Kit (Qiagen, Venlo, The Netherlands) according to the manufacturer’s instructions, and 1 μg of total RNA was reverse transcribed using High-Capacity cDNA Reverse Transcription Kit (Thermo Scientific). Gene expression was assessed using The TaqMan® Gene Expression Master Mix and the assays Hs_01085704g1, Hs00155426m1, Hs00608023_m1, and Hs00236329_m1, using the 7300 real-time PCR system (Applied Biosystems, Foster City, CA, USA). Results were normalized to the level of the ubiquitously expressed RNA 18S ribosomal 1 gene (RN18S, Hs03928990_g1), and the Universal Human Reference RNA (Agilent) was used as control. RNA 18S ribosomal 1 gene was used as a internal control gene (RN18S, Hs03928990_g1). Results were expressed as 2^−ΔCt^ (ΔCt = [(CT gene of interest − CT internal control) to compare the relative gene expression among samples, and as 2^−ΔΔCt^ (ΔΔCt = [(CT gene of interest − CT internal control) sample − (CT gene of interest − CT internal control) universal]) to compare gene expression of the treated cell lines with that of untreated control [63].

### Flow cytometric analysis of P-gp expression and P-gp direct dye efflux assay

These were performed essentially as described [64].

### Co-culture with human HS-5 stromal cells

HS-5 stromal cells were grown in the lower chamber of Transwell® six-well plates (Corning, New York, NY, USA) containing a 0.4 μm of polyester membrane, then T-ALL cells (375,000) were added to the upper chamber and treated with nelarabine (2–5 μM). After 48 h, the viability of treated cell lines grown either alone or co-cultured was evaluated. Cells were collected and analyzed for further experiments.

### Combined drug effect analysis

The combination effect and a potential synergy were evaluated from quantitative analysis of dose-effect relationships as described previously [62]. For each combination experiment, a combination index (CI) number was calculated using the Biosoft CalcuSyn software (Cambridge, UK).

### Flow cytometric analysis of p-AKT (Ser473), p-ERK (Thr202), and Bcl2 levels in T-ALL patient samples

Blasts from pediatric patients with T-ALL were fixed/permeabilized in methanol 90 % for at least 30 min, then washed in PBS with 4 % FBS. Cells were incubated with primary antibodies conjugated to AlexaFluor® 488 or 647, from Cell Signaling. Isotype controls were employed as well. Cells were analyzed on a FC500 flow cytometer (Beckman Coulter) with the appropriate software (CXP, Beckman Coulter). At least 15,000 events per sample were acquired. Absolute cell count by flow cytometry was performed using eBeads123 (Affimetrix eBiosciences, Santa Clara, CA, USA).

### Statistical analysis

The data are presented as mean values from three separate experiments ± SD. Significant effects between treatment and control groups in the in vitro studies were analyzed using Student’s unpaired *t* test. Mann–Whitney test was used to statistically analyze the differences in the two subgroups of sensitive/resistant to nelarabine T-ALL cells.

## Additional files

Additional file 1: Figure S1. DNA damage and ROS production. A. Flow cytometric analysis of γH2AX in MOLT-4 and Jurkat sensitive to nelarabine cells (upper panel). Percentages of γH2AX are shown in the table (lower panel). B. Reactive oxygen species (ROS) production was assessed in MOLT-4 and Jurkat sensitive T-ALL cell lines after 1 and 24 h treatment with nelarabine. Fluorescence values (DCF) were reported as the percentage of intracellular ROS in respect to controls. ROS increased significantly in both 1- and 24-h treatment compared to controls in MOLT-4 and Jurkat cells. Statistical analyses were performed with the Dunnett’s multiple comparison test.
Additional file 2: Figure S2. Expression of dCK and dGK in T-ALL cell lines. Western blotting analyses for the expression of dCK and dGK proteins in T-ALL cell lines. Fifty micrograms of protein was blotted to each lane. Antibody to β-actin served as a loading control. Molecular weights are indicated on the right. One representative of two different experiments is shown.
Additional file 3: Figure S3. Bcl2 expression in T-ALL cell lines. Western blotting analyses for the basal expression of Bcl2 in untreated T-ALL cell lines. Twenty micrograms of protein was blotted to each lane. Antibody to β-actin served as a loading control. Molecular weights are indicated on the right. Densitometric analysis was performed using a Chemidoc 810 Imager with the appropriate software (UVP, Upland, CA, USA), and for each cell line, Bcl2 protein expression is indicated relatively to β-actin expression.
Additional file 4: Figure S4. The combination of nelarabine and ZSTK-474 is synergistic in CEM-R cells, which overexpress P-gp. Cell viability assay of CEM-R cell line treated for 48 h with increasing concentrations of nelarabine alone or combined with the pan PI3K p110 inhibitor ZSTK-474. One representative of two different experiments is shown.
Additional file 5: Figure S5. Specific effects of nelarabine on PI3K/AKT and MEK/ERK1/2 pathways. Western blotting analyses for the expression of p-AKT and p-ERK in resistant T-ALL cell lines treated with the specific inhibitors LY294002 (PI3K inhibitor), CCI-779 (mTOR allosteric inhibitor) or trametinib (MEK1/2 inhibitor) alone or in combination with nelarabine. Thirty micrograms of protein was blotted to each lane. Antibody to β-actin served as a loading control. Molecular weights are indicated on the right. CTRL: untreated cells; Nela and N: nelarabine at 10 μM; LY: LY294002 at 10 μM; CCI: CCI-779 at 100 nM; Tram: trametinib at 1 μM. Cells were treated for 48 h.
Additional file 6: Figure S6. HS-5 stromal cells protect MOLT-4 cells from nelarabine cytotoxicity at least in part through CXCL12/CXCR4 interactions. A. MTT assays of MOLT-4 cells growing alone or in co-culture system with HS-5 cells and treated with nelarabine (2 μM for 48 h) in a Transwell^@^ system. Three hundred seventy-five thousand MOLT-4 cells were seeded in the upper chamber of the Transwell^@^ system. Results are the mean of three different experiments ± SD. B. Western blot analysis for p-ERK, p-CXCR4, and their total forms in MOLT-4 cells grown either in the absence or the presence of HS-5 stromal cells in a Transwell^@^ system. Nelarabine treatment (2 μM) was for 24 h. Fifty micrograms of protein was blotted to each lane. Antibody to β-actin served as a loading control. Molecular weights are indicated on the right. One representative of two different experiments is shown.

## Abbreviations

AML: Acute myeloid leukemia
Bak: Bcl-2 homologous antagonist/killer
Bax: BCL2 associated X protein
Bcl2: B cell lymphoma 2
B-CLL: B cell chronic lymphocytic leukemia
Bcl-xL: B cell lymphoma-extra large
BM: Bone marrow
CEM-R: CEM overexpressing P-gp
CI: Combination index
CR: Complete remission
CVAD: Cyclophosphamide, vincristine, doxorubicin, dexamethasone
CXCL12: Chemokine C-X-C motif ligand 12
CXCR4: Chemokine C-X-C motif receptor 4
dCK: Deoxycytidine kinase
dGK: Deoxyguanosine kinase
ENT1/2: Equilibrative nucleoside transporters1/2
ETP-ALL: Early T precursor acute lymphoblastic leukemia
HS-5: Human stromal cells 5
Mcl1: Myeloid cell leukemia 1
MEK: Mitogen-activated protein kinase kinase
mTOR: Mammalian target of rapamycin
PARP: Poly(ADP-ribose) polymerase
PCR: Polymerase chain reaction
P-gp: P-glycoprotein
PI: Propidium iodide
PI3K: Phosphoinositide 3-kinase
S6RP: S6 ribosomal protein
T-ALL: T cell acute lymphoblastic leukemia
T-LL: T cell lymphocytic lymphoma
