# Supplementary figures and images for: Improving nelarabine efficacy in T cell acute lymphoblastic leukemia by targeting aberrant PI3K/AKT/mTOR signaling pathway
Source: J Hematol Oncol. 2016 Oct 24;9:114. doi: 10.1186/s13045-016-0344-4 (PMC5075755; doi:10.1186/s13045-016-0344-4)

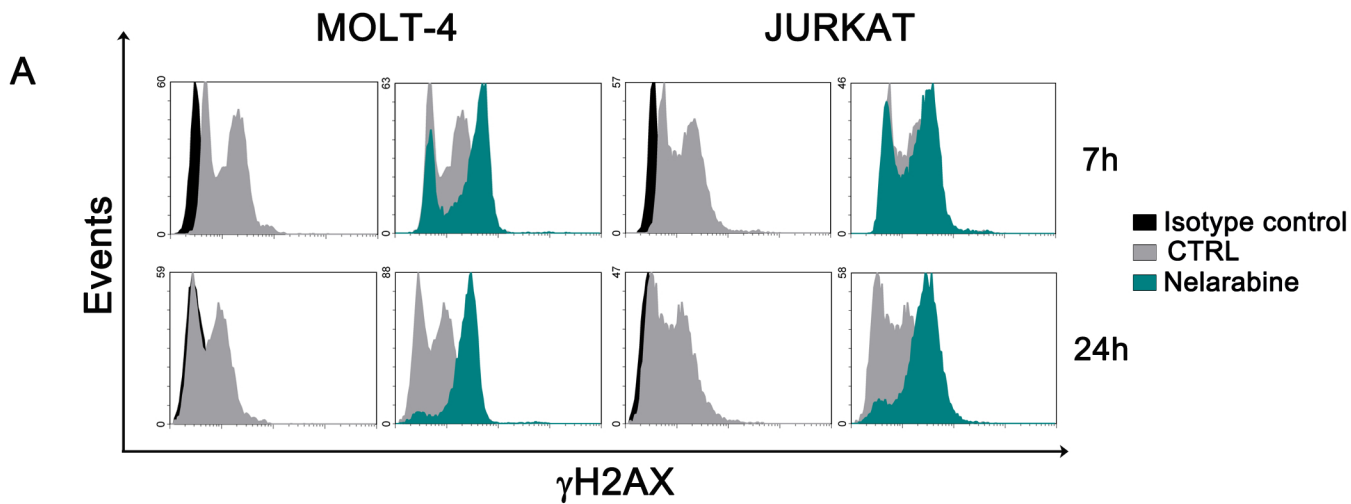

|           | MOLT-4 |       | JURKAT |       |
|-----------|--------|-------|--------|-------|
| treatment | CTRL   | NELA  | CTRL   | NELA  |
| 7h        | 62.41  | 74.21 | 64.79  | 70.59 |
| 24h       | 55.96  | 93.49 | 54.77  | 90.61 |

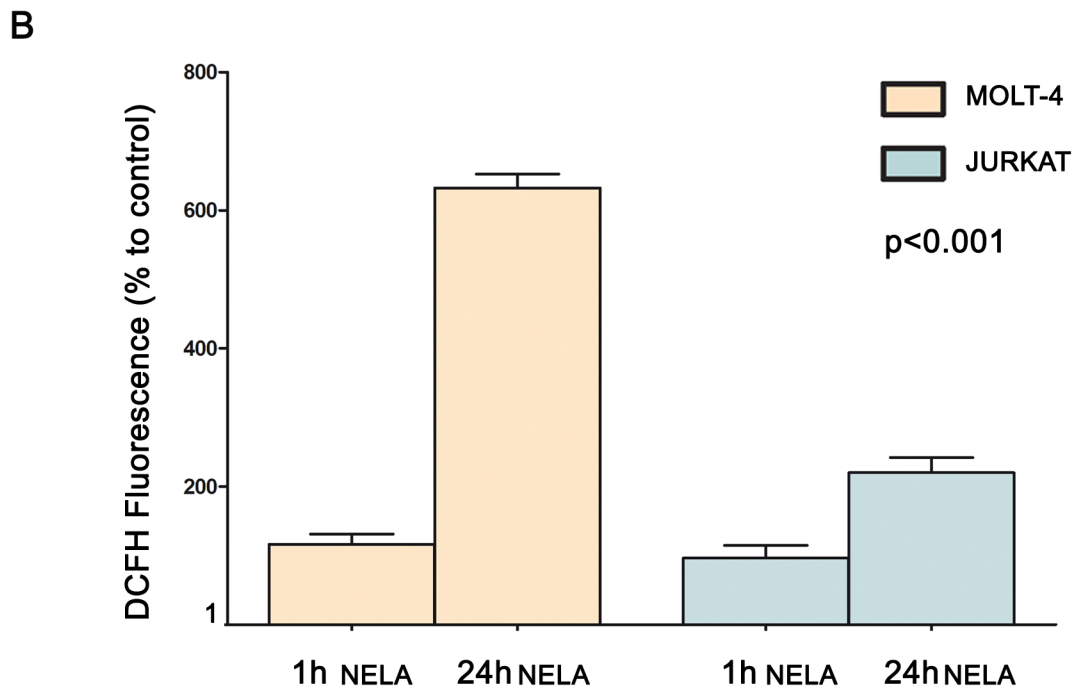

**Additional fig. 1**

Supplement: Additional file 1: Figure S1. — DNA damage and ROS production. A. Flow cytometric analysis of γH2AX in MOLT-4 and Jurkat sensitive to nelarabine cells (upper panel). Percentages of γH2AX are shown in the table (lower panel). B. Reactive oxygen species (ROS) production was assessed in MOLT-4 and Jurkat sensitive T-ALL cell lines after 1 and 24 h treatment with nelarabine. Fluorescence values (DCF) were reported as the percentage of intracellular ROS in respect to controls. ROS increased significantly in both 1- and 24-h treatment compared to controls in MOLT-4 and Jurkat cells. Statistical analyses were performed with the Dunnett’s multiple comparison test. [file 13045_2016_344_MOESM1_ESM.pdf]

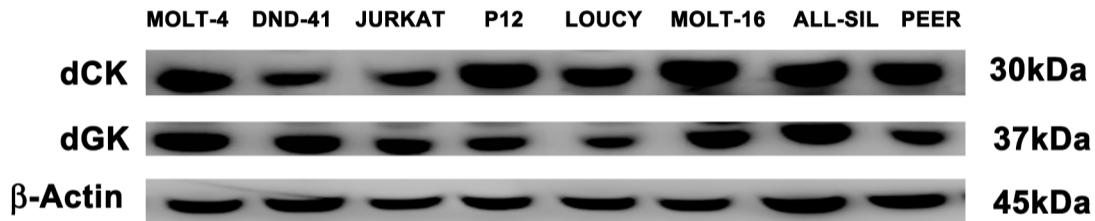

**Additional fig 2**

Supplement: Additional file 2: Figure S2. — Expression of dCK and dGK in T-ALL cell lines. Western blotting analyses for the expression of dCK and dGK proteins in T-ALL cell lines. Fifty micrograms of protein was blotted to each lane. Antibody to β-actin served as a loading control. Molecular weights are indicated on the right. One representative of two different experiments is shown. [file 13045_2016_344_MOESM2_ESM.pdf]

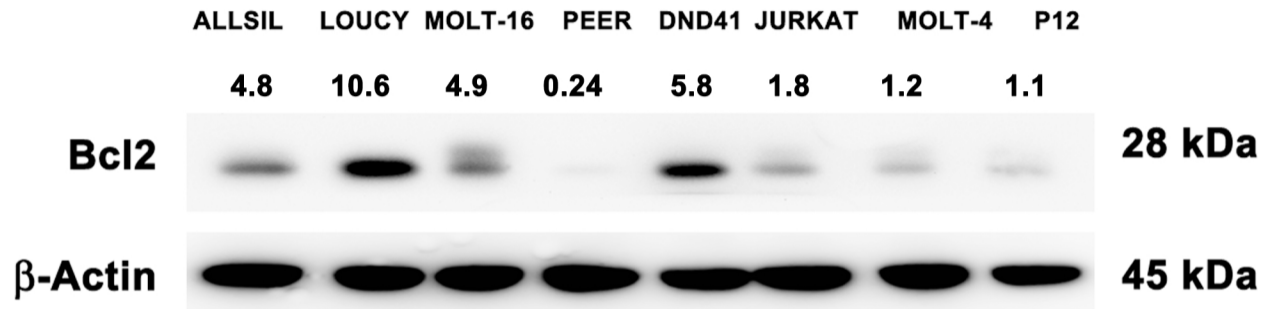

**Additional fig. 3**

Supplement: Additional file 3: Figure S3. — Bcl2 expression in T-ALL cell lines. Western blotting analyses for the basal expression of Bcl2 in untreated T-ALL cell lines. Twenty micrograms of protein was blotted to each lane. Antibody to β-actin served as a loading control. Molecular weights are indicated on the right. Densitometric analysis was performed using a Chemidoc 810 Imager with the appropriate software (UVP, Upland, CA, USA), and for each cell line, Bcl2 protein expression is indicated relatively to β-actin expression. [file 13045_2016_344_MOESM3_ESM.pdf]

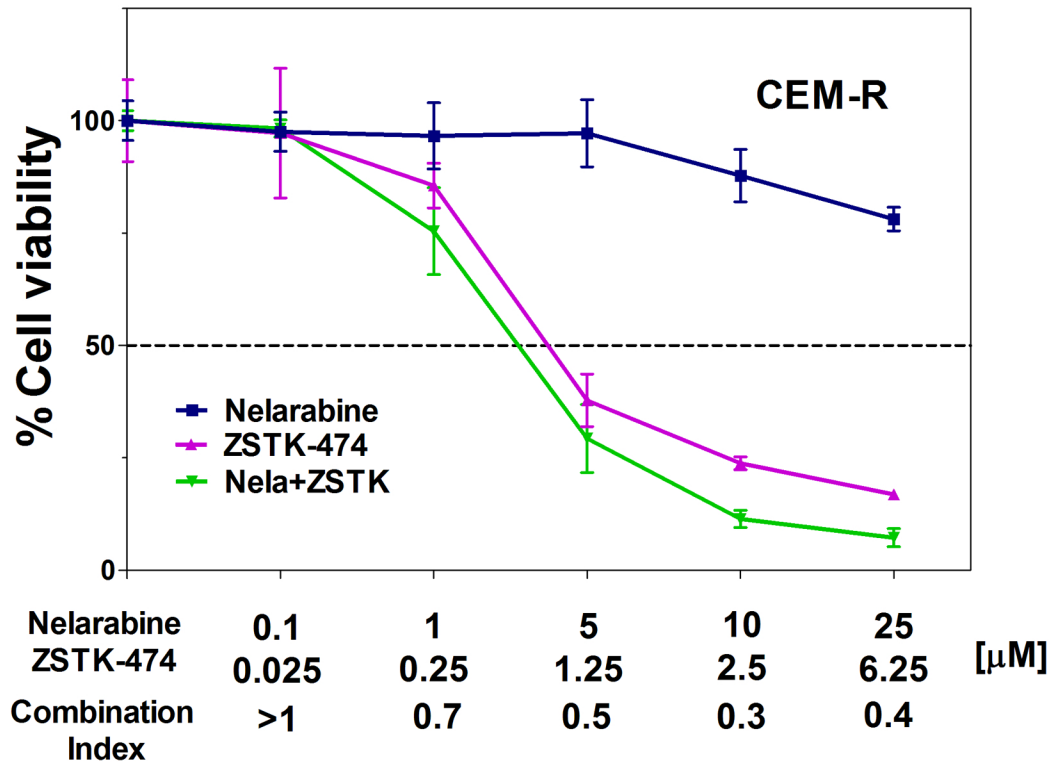

**Additional fig 4**

Supplement: Additional file 4: Figure S4. — The combination of nelarabine and ZSTK-474 is synergistic in CEM-R cells, which overexpress P-gp. Cell viability assay of CEM-R cell line treated for 48 h with increasing concentrations of nelarabine alone or combined with the pan PI3K p110 inhibitor ZSTK-474. One representative of two different experiments is shown. [file 13045_2016_344_MOESM4_ESM.pdf]

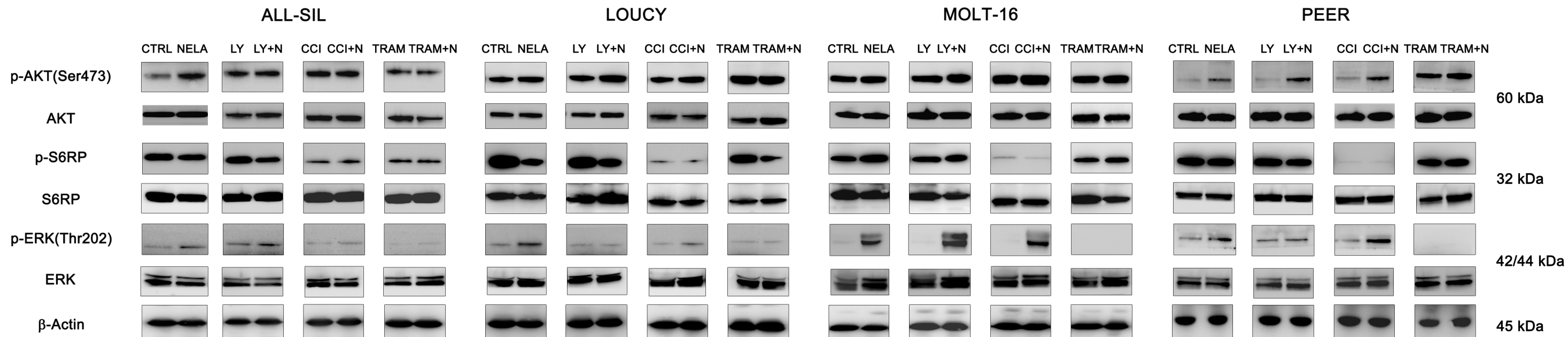

Additional fig 5

Supplement: Additional file 5: Figure S5. — Specific effects of nelarabine on PI3K/AKT and MEK/ERK1/2 pathways. Western blotting analyses for the expression of p-AKT and p-ERK in resistant T-ALL cell lines treated with the specific inhibitors LY294002 (PI3K inhibitor), CCI-779 (mTOR allosteric inhibitor) or trametinib (MEK1/2 inhibitor) alone or in combination with nelarabine. Thirty micrograms of protein was blotted to each lane. Antibody to β-actin served as a loading control. Molecular weights are indicated on the right. CTRL: untreated cells; Nela and N: nelarabine at 10 μM; LY: LY294002 at 10 μM; CCI: CCI-779 at 100 nM; Tram: trametinib at 1 μM. Cells were treated for 48 h. [file 13045_2016_344_MOESM5_ESM.pdf]

**A**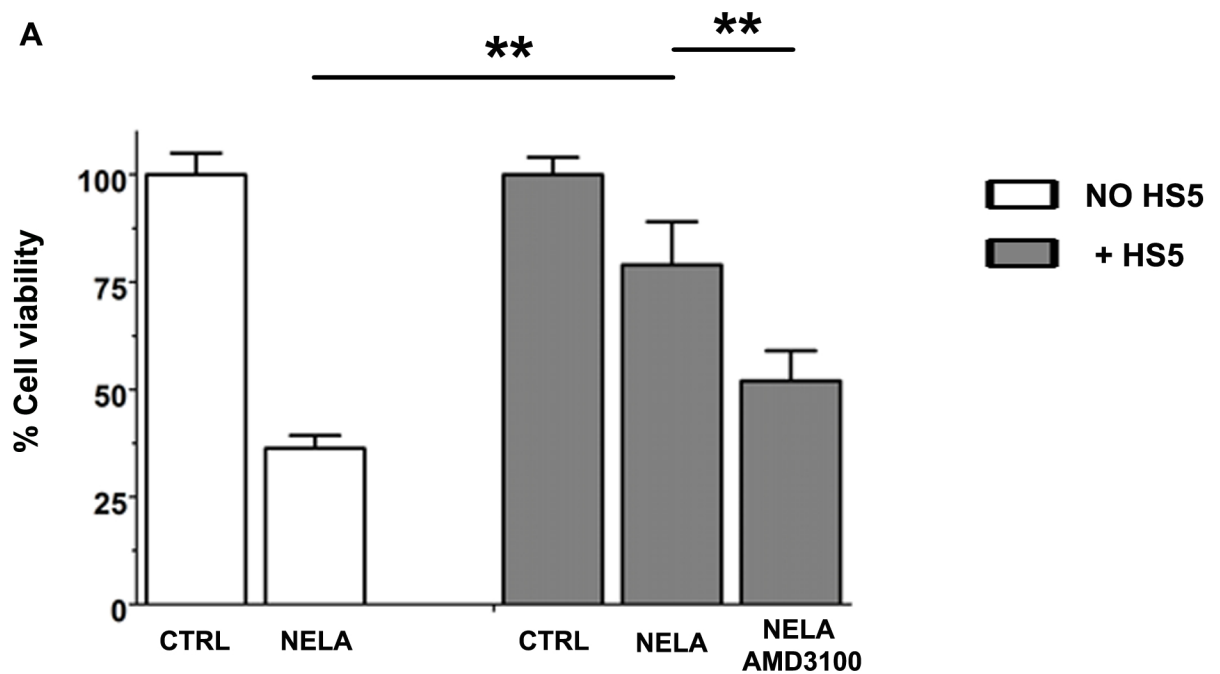**B**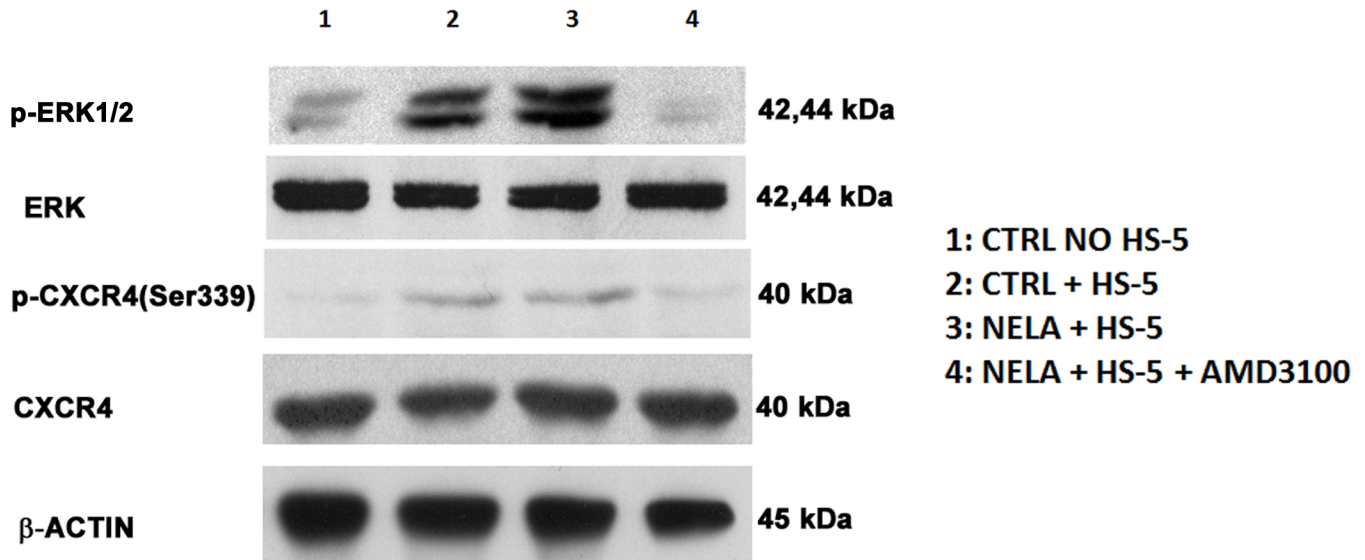

Supplement: Additional file 6: Figure S6. — HS-5 stromal cells protect MOLT-4 cells from nelarabine cytotoxicity at least in part through CXCL12/CXCR4 interactions. A. MTT assays of MOLT-4 cells growing alone or in co-culture system with HS-5 cells and treated with nelarabine (2 μM for 48 h) in a Transwell@ system. Three hundred seventy-five thousand MOLT-4 cells were seeded in the upper chamber of the Transwell@ system. Results are the mean of three different experiments ± SD. B. Western blot analysis for p-ERK, p-CXCR4, and their total forms in MOLT-4 cells grown either in the absence or the presence of HS-5 stromal cells in a Transwell@ system. Nelarabine treatment (2 μM) was for 24 h. Fifty micrograms of protein was blotted to each lane. Antibody to β-actin served as a loading control. Molecular weights are indicated on the right. One representative of two different experiments is shown. [file 13045_2016_344_MOESM6_ESM.pdf]
